# Autoimmune encephalitis in a resource-limited public health setting: a case series analysis

**DOI:** 10.1055/s-0044-1779054

**Published:** 2024-02-07

**Authors:** Matheus Bernardon Morillos, Wyllians Vendramini Borelli, Giovani Noll, Cristian Daniel Piccini, Martim Bravo Leite, Alessandro Finkelsztejn, Marino Muxfeldt Bianchin, Raphael Machado Castilhos, Carolina Machado Torres

**Affiliations:** 1 Hospital de Clínicas de Porto Alegre, Serviço de Neurologia, Porto Alegre RS, Brazil.; 2 Universidade Federal do Rio Grande do Sul, Faculdade de Medicina, Porto Alegre RS, Brazil.; 3 Universidade Federal do Rio Grande do Sul, Faculdade de Medicina, Departamento de Medicina Interna, Porto Alegre RS, Brazil.; 4 Universidade Federal do Rio Grande do Sul, Faculdade de Medicina, Programa de Pós-Graduação em Medicina: Ciências Médicas, Porto Alegre RS, Brazil.

**Keywords:** Autoantibodies, Seizures, Paraneoplastic Syndromes, Nervous System, Limbic Encephalitis, Autoanticorpos, Convulsões, Síndromes Paraneoplásicas do Sistema Nervoso, Encefalite Límbica

## Abstract

**Background** Autoimmune encephalitis (AE) consists of a group of acquired diseases that affect the central nervous system. A myriad of phenotypes may be present at the onset. Due to the heterogeneity of clinical presentations, it is difficult to achieve uniformity for the diagnostic and therapeutic processes and follow-up strategies.

**Objective** To describe a series of patients diagnosed with AE in a resource-limited public hospital in southern Brazil and to analyze therapeutics and outcomes.

**Methods** We retrospectively reviewed the electronic medical records of patients diagnosed with AE at the Hospital de Clínicas de Porto Alegre from 2014 to 2022. Data collected included clinical presentation, neuroimaging, cerebrospinal fluid testings, electroencephalogram, autoantibodies, treatments, outcomes, follow-up time, degree of neurological impairment, and mortality.

**Results** Data from 17 patients were retrieved. Eleven cases were classified as definite AE and 6 as possible AE. Autoantibodies were identified in 9 patients. Timing for diagnosis was impacted by the high costs associated with autoantibody testing. Most patients became functionally dependent (82.4%) and most survivors remained with autoimmune-associated epilepsy (75%). Five patients died during hospitalization, and one after a 26-month of follow-up.

**Conclusion** In this resource-limited hospital, patients with AE had a worse clinical outcome than that previously described in the literature. Development of epilepsy during follow-up and mortality were greater, whilst functional outcome was inferior. Autoantibody testing was initially denied in most patients, which impacted the definitive diagnosis and the use of second-line therapies.

## Resumo

**Antecedentes** A encefalite autoimune (EA) consiste em um grupo de doenças adquiridas que afetam o sistema nervoso central.

**Objetivo** Descrever uma série de pacientes diagnosticados com EA em um contexto de atenção terciária à saúde com recursos limitados e analisar a terapêutica e os resultados.

**Métodos** Revisamos retrospectivamente os prontuários eletrônicos de pacientes diagnosticados com EA no Hospital de Clínicas de Porto Alegre de 2014 a 2022. Os dados coletados incluíram apresentação clínica, neuroimagem, exames de líquido cefalorraquidiano, eletroencefalograma, autoanticorpos, tratamentos, resultados, tempo de acompanhamento, grau de comprometimento neurológico e mortalidade.

**Resultados** Dados de 17 pacientes foram coletados. Onze casos foram classificados como EA definitivo e seis como EA possível. Autoanticorpos foram identificados em nove pacientes. O tempo para o diagnóstico foi afetado pelos altos custos associados ao teste de autoanticorpos. A maioria dos pacientes tornou-se funcionalmente dependente (82,4%), e a maioria dos sobreviventes permaneceu com epilepsia autoimune associada (75%). Cinco pacientes faleceram durante a internação, e um após 26 meses de seguimento.

**Conclusão** No hospital em questão, os pacientes com EA tiveram um desfecho clínico pior do que o previamente descrito na literatura. O desenvolvimento de epilepsia durante o acompanhamento e a mortalidade foram maiores, enquanto o desfecho funcional foi inferior. Os testes de autoanticorpos foram inicialmente negados para a maioria dos pacientes, o que impactou o diagnóstico definitivo e o uso de terapias de segunda linha.

## INTRODUCTION

Autoimmune encephalitis (AE) comprises a group of inflammatory diseases of the central nervous system (CNS) with great clinical and prognostic heterogeneity. 1 With the recent improvement in the identification of autoantibodies, new studies have suggested a prevalence that is comparable to infectious encephalitis. 2 Clinical presentation is varied and predominantly includes epileptic seizures, cognitive impairment, and psychiatric symptoms. 3 As a serious, progressive, and debilitating disease, delayed diagnosis can lead to severe functional impairment or death. 4

Early diagnosis and management are essential for better outcomes. Serum or cerebrospinal fluid (CSF) detection of specific autoantibodies is possible in many cases, but these exams are not always performed due to several reasons. 1 5 First, autoantibodies are not always available. 6 Second, if available, these and other complementary exams, such as magnetic resonance imaging (MRI), tend to be expensive. Some healthcare sectors have reported restrictions and financial difficulties, making diagnosis difficult. 7 Resource-limited settings are particularly vulnerable to high-cost exams and may potentially overlook the majority of cases of AE. 8 Finally, the myriad of clinical symptoms associated with low clinical suspicion makes it harder to early diagnose this condition and initiate a therapy that could avoid negative outcomes. Up to this date, few studies have been conducted in developing countries and despite the increasing incidence and comprehension of AE, this condition remains poorly understood. 9

Here, we describe several cases of AE, their therapeutic response, clinical features, and outcomes in a tertiary referral hospital in the South Region of Brazil. We aim to analyze the challenges in diagnosing and treating these individuals, as well as identify the main forms of AE in this region. As it will be shown, a higher-than-expected proportion of cases had a worse outcome, which may be related to the identification of more patients with autoantibodies against intracellular antigens, but also due to the difficulties related to late diagnosis and treatment.

## METHODS

This is a case series study of patients diagnosed with AE at Hospital de Clínicas de Porto Alegre (HCPA), a public tertiary hospital in Brazil. This study was approved by the research ethics committee of the Hospital de Clínicas de Porto Alegre (HCPA) and registered in *Brazil Platform* under Certificate of Presentation for Ethical Appreciation (CAAE, in Portuguese abbreviation) number 63246722.5.0000.5327. Since this is a retrospective observational study and all data provided, including images, is anonymized, HCPA waived informed consent.

We selected patients with a diagnosis of possible, probable, or definite AE from 2014 to 2022, according to Graus et al.'s criteria. 5 The patients selected for this study had been registered in a database of the Neurology Service of the HCPA, which has existed since 2014, for cases assessed as AE at the time. All patients who did not die during hospitalization were followed up on an outpatient basis at our service. This study follows the CARE Case Report Guidelines, 10 adapted to the context of a case series study.

In order to characterize AE, other etiologies were necessarily excluded. These include systemic or CNS infection, metabolic encephalopathy, drug or illicit drug toxicity, cerebrovascular disease, vasculitis, neoplasia, rheumatological, and psychiatric disorders. Afterwards, following the criteria of Graus et al., 5 the patient was evaluated as having either possible or probable AE if the auto-antibody was unknown or not identified. To be diagnosed as having possible AE, the patient had to present subacute changes in mental state or behavior, or loss of working memory, and at least one of the following: related focal involvement of the CNS, epileptic seizure without a previous history of epilepsy, pleocytosis in CSF analysis or suggestive findings in MRI examination. To be diagnosed as having probable AE, on the other hand, specific clinical syndrome or radiological findings, such as acute disseminated encephalomyelitis (ADEM), Bickerstaff's brainstem encephalitis, limbic encephalitis, or anti-NMDA receptor encephalitis, had to be identified. Furthermore, a patient could be classified as having probable AE after exclusion of all known specific clinical syndromes and after no CSF or serum auto-antibodies were identified, as long as they met the diagnostic criteria for Hashimoto's encephalopathy or autoantibody-negative but probable AE. A diagnosis of Hashimoto's encephalopathy could be made when all of the following criteria were met: encephalopathy accompanied by epileptic seizures, myoclonus, hallucinations, or stroke-like episodes; thyroid dysfunction; normal MRI findings; presence of thyroid-related antibodies such as antibodies against thyroid peroxidase (TPOAb) and thyroglobulin (TgAb); absence of neuronal antibodies; and exclusion of alternative causes. For the diagnosis of autoantibody-negative but probable AE, in addition to the exclusion of alternative plausible diagnoses, the patient had to present with specific clinical syndromes, non-identification of serum or CSF autoantibodies, altered mental status, psychiatric symptoms, or subacute onset of working memory deficit, plus two of the following: MRI findings suggestive of AE, CSF pleocytosis, or cerebrospinal fluid-specific oligoclonal bands, and brain biopsy demonstrating inflammatory infiltrates. When a specific auto-antibody was identified in a context of clinical suspicion, the patient was considered as having definite AE. However, in some disorders, the clinical syndrome and radiological findings allow for the classification of definite AE even before knowing the antibody status. 5 This way, it was possible to diagnose definite autoimmune limbic encephalitis—for instance—if, in addition to subacute changes in mental state, behavior or working memory, we identified the presence of high signal on T2-weighted FLAIR MRI highly restricted to the mesial structures of both temporal lobes, as well as the presence of at least one of the following: pleocytosis in CSF analysis or irritative electroencephalographic changes involving the temporal lobes.

Electronic medical reports were retrieved for demographic, clinical, and ancillary testing data using a standardized form. Data collection included clinical symptoms and ancillary testing collected were MRI, electroencephalogram (EEG), and laboratory exam (blood and CSF) findings at the moment of diagnosis. MRI images were retrieved and analyzed by a neuroradiologist and a neurologist with clinical expertise. The modified Rankin Scale (mRS) was used to categorize functional outcomes, and patients' follow-up was collected in outpatient clinics consultations. Categorical variables were described as frequencies and proportions, and continuous variables as percentages and mean.

Noteworthy, data was collected from a public tertiary hospital, which is a major component of the national public health system. In this context, high-cost exams, such as the evaluation of serum autoantibodies, must be evaluated by the financial staff initially which determines the approval or refusal of such an exam according to the degree of clinical suspicion. When approved, the biological sample is directed to an external laboratory to perform the exam. Prior authorizations are not routinely performed in high-cost exams.

To detect anti-GAD serum autoantibody, enzyme-linked immunosorbent assay (ELISA) was used; it was only considered significant when in high titers (> 1000 IU/ml). For anti-GAD, anti-NMDAr, anti-AQP4, and anti-VGKC autoantibodies detection in the CSF, cell-based assay (CBA) technique utilizing indirect immunofluorescence (IIF) was used. Finally, to detect serum autoantibodies anti-Ma2, anti-HU, and anti-YO, the immunoblotting technique was used. All autoantibodies, except for serum anti-GAD, were considered significant when above the detection reference value in an appropriate clinical context.

## RESULTS

Our sample comprises 17 patients, with a high proportion of Caucasians (88%) and a similar distribution between genders - as shown in Supplementary Material ( https://www.arquivosdeneuropsiquiatria.org/wp-content/uploads/2023/10/ANP-2023.0136-Supplementary-Material.xlsx ). The mean age was 36.2 [ ± 19.8] years and the mean years of schooling was 8.8 [ ± 3.92]. From these 17 encephalitis cases, 11 met the criteria for definite AE, of which 9 were antibody-positive and 2 were negative; 6 met the criteria for possible AE. A detailed characterization of the diagnostic criteria met by each patient can be seen in Tables 1 , 2 , and 3 .

**Table 1:** Patients who fulfilled the diagnostic criteria for definite anti-NMDA receptor encephalitis

Patient number	Clinical manifestations*	Anti-NMDAr IgG antibodies identification in CSF	Reasonable exclusion of alternative causes	Definite AE
2	AB + S	Yes	Yes	Yes
3	AB + S	Yes	Yes	Yes
4	AB + DLC + S	Yes	Yes	Yes

Abbreviations: AE, autoimmune encephalitis; S, seizures; AB, abnormal behaviour; DLC, decreased level of consciousness; CSF, cerebrospinal fluid; anti-NMDAr, anti-N-methyl-d-aspartate-receptor.

Notes: *According to Graus et al., to be diagnosed with definite anti-NMDA receptor encephalitis, the patient has to present rapid onset of one or more symptoms of the following: 1) Abnormal behavior or cognitive dysfunction; 2) Speech dysfunction; 3) Seizures; 4) Movement disorder, dyskinesias, or rigidity/abdnormal postures; 5) Decreased level of consciouness; 6) Autonomic dysfunction or central hypoventilation.

**Table 2:** Patients who fulfilled the diagnostic criteria for definite autoimmune limbic encephalitis

Patient number	Subacute onset of WM, S or PS	Bilateral brain abdnormalities on T2-weighted fluid-attenuated inversion recovery MRI highly restricted to the medial temporal lobes	CSF pleocytosis or EEG with epileptic or slow-wave activity involving the temporal lobes	Antibodies identification	Reasonable exclusion of alternative causes	Definite AE
5	WM + PS	Yes	CSF pleocytosis	Anti-GAD/Anti-VGKC	Yes	Yes
6	PS	Yes	CSF pleocytosis + EEG with SWA-TL	Anti-GAD	Yes	Yes
7	S + PS	Yes	CSF pleocytosis	No	Yes	Yes
8	S + PS	Yes	CSF pleocytosis	No	Yes	Yes
9	PS	Yes	CSF pleocytosis	Anti-GAD	Yes	Yes
15	S + PS	Yes	EEG with epileptic activity in the TL	Anti-GAD	Yes	Yes

Abbreviations: AE, autoimmune encephalitis; PS, psychiatric symptoms; S, seizures; WM, working memory deficits; CSF, cerebrospinal fluid; EEG, electroencephalography; SWA, slow-wave activity; TL, temporal lobes; MRI, magnetic resonance imaging; anti-GAD, anti-glutamic acid decarboxylase; anti-VGKC, anti-voltage-gated potassium channel complex antibodies.

**Table 3:** Patients who fulfilled the diagnostic criteria for definite autoimmune encephalitis with detection of specific autoantibodies or criteria for possible autoimmune encephalitis

Patient number	Subacute onset of WM, AM or PS	New focal CNS finding, S not explained by a previously known seizure disorder, CSF pleocytosis, or MRI features suggestive of encephalitis	Antibody identification	Reasonable exclusion of alternative causes	Definite AE	Possible AE
1	AM + PS	CSF pleocytosis + suggestive MRI*	Anti-Ma2	Yes	X	
13	WM + AM + PS	CSF pleocytosis + suggestive MRI**	Anti-HU	Yes	X	
10	AM + PS	CSF pleocytosis	No	Yes		X
11	WM + PS	CSF pleocytosis + S + suggestive MRI***	No	Yes		X
12	WM + AM + PS	CSF pleocytosis + S + suggestive MRI***	No	Yes		X
14	AM + PS	S	No	Yes		X
16	WM	CSF pleocytosis + S + suggestive MRI***	No	Yes		X
17	AM	CSF pleocytosis + focal CNS finding + suggestive MRI***	No	Yes		X

Abbreviations: AE, autoimmune encephalitis; AM, altered mental status; PS, psychiatric symptoms; S, seizures; WM, working memory deficits; CNS, Central nervous system; CSF, Cerebrospinal fluid; MRI, magnetic resonance imaging; anti-Ma2, antineuronal antibody against Ma2 antigen; anti-Hu, anti-neuronal nuclear antibodies type 1.

Notes: *Temporal lobes and diencephalon involvement; **Temporal lobe, diencephalon and basal ganglia involvement; ***Temporal lobe, insula and basal ganglia involvement.

Although antibodies were ordered for all patients, requests were not uniform and not all were granted ( Table 4 ). Among patients with unidentified autoantibodies, 6 were classified as having possible AE (patients 10, 11, 12, 14, 16, and 17) and 2 as having definite autoimmune limbic encephalitis without autoantibody identification (patients 7 and 8). We consider it more prudent to classify the former as having possible AE rather than negative autoantibody but probable AE because we cannot rule out that autoantibodies for which testing was not authorized could be present in these patients.

**Table 4:** Ordered and authorized autoantibodies and time interval until testing

Patient number	Ordered AA	Authorized AA	Days until authorization
	Anti-Ma1; Anti-Ma2; Anti-Hu; Anti-CV2/CRMP5	Anti-Ma2	28
2	Anti-NMDAr; Anti-Hu; Anti-Ma1; Anti-Ma2; Anti-Yo; Anti-LG1; Anti-CV2/CRMP5; Anti-Gaba-A; Anti-GabaB	Anti-NMDAr*	1
3	Anti-NMDAr	Anti-NMDAr*	1
4	Anti-NMDAr	Anti-NMDAr	11
5	Anti-NMDAr; Anti-Ma2; Anti-Hu; Anti-GAD; Anti-VGKC	Anti-NMDAr; Anti-Ma2; Anti-Hu; Anti-GAD; Anti-VGKC	13
6	Anti-NMDAr; Anti-GAD	Anti-NMDAr; Anti-GAD	12
7	Anti-Hu; Anti-NMDAr	Anti-NMDAr	1
8	Anti-NMDAr; Anti-Hu; Anti-LG1; Anti-CV2/CRMP5; Anti-Gaba-A; Anti-GabaB; Anti-CASPR2	Anti-Hu; Anti-NMDAr	5
9	Anti-NMDAr; Anti-Hu; Anti-GAD	Anti-NMDAr; Anti-Hu; Anti-GAD	1
10**	Not available	Not available	**-**
11	Anti-NMDAr; Anti-Hu; Anti-GAD; Anti-VGKC; Anti-GabaA; Anti-GabaB	Not authorized	**-**
12	Anti-NMDAr; Anti-Hu	Anti-NMDAr	7
13	Anti-NMDAr; Anti-Hu; Anti-Yo; Anti-GAD	Anti-NMDAr; Anti-Hu; Anti-Yo; Anti-GAD	9
14	Anti-NMDAr; Anti-Hu; Anti-VGKC; Anti-AMPAr; Anti-Gaba-A; Anti-GabaB; Anti-CASPR2; AntiAQP4	Anti-NMDAr; Anti-GAD; Anti-AQP4	1
15	Anti-NMDAr; Anti-AMPAr; Anti-Gaba-A; Anti-GabaB; Anti-CASPR2	Not authorized***	**-**
16	Anti-Hu; Anti-GabaB	Not authorized	−
17	Anti-NMDAr; Anti-Hu; Anti-Yo	Anti-Hu; Anti-Yo	3

Abbreviations: Aa, auto-antibody; anti-Ma1, antineuronal antibody against Ma1 antigen; anti-Ma2, antineuronal antibody against Ma2 antigen; anti-Hu, anti-neuronal nuclear antibodies type 1; anti-CV2/CRMP5, anti-collapsin response mediator protein 5 antibody; anti-NMDAr, anti-N-methyl-d-aspartate-receptor; anti-Yo, anti-Purkinje-cell cytoplasmic autoantibody type 1; anti-LG1, anti-leucine-rich glioma inactivated 1; anti-GabaA, auto-antibody against γ-aminobutyric acid-A receptor; anti-GabaB, auto-antibody against γ-aminobutyric acid-B receptor; anti-GAD, anti-glutamic acid decarboxylase; anti-VGKC, anti-voltage-gated potassium channel complex antibodies; anti-CASPR2, anti-contactin-associated protein-like 2 antibody; anti-AMPAr, antibody against alpha-amino-3-hydroxy-5-methyl-4-isoxazolepropionic acid receptor; anti-AQP4, anti-aquaporin-4.

Notes: *Private funding; **AE not suspected during hospitalization; ***Patient previously known as anti-GAD positive.

Behavioral changes were the most common initial manifestation (9 patients, 53%), followed by epileptic seizures (5 patients, 29.4%). The time frame from the onset of symptoms to the hospitalization that led to diagnosis ranged from 0 to 120 days, with a mean value of 28 [ ± 36.8] days. Patients presenting with epileptic seizures were hospitalized significantly earlier compared to those with other clinical presentations. In this series, 4 patients (patients 2, 3, 14, and 16) were initially managed in a psychiatric inpatient unit with a presumed diagnosis of psychosis (2 patients), mania (1 patient), and depression (1 patient). Three of them died during hospitalization.

Most patients were initially treated with methylprednisolone 1g intravenously for 3 to 5 days (15 patients, 88.2%). The majority of these patients (n = 12) received some additional immunomodulatory therapy based on clinical judgment, therapeutic availability, and disease severity ( Supplementary Material ). Only 2 patients did not receive any treatment, one because of a suspected underlying bacterial infection and the other due to low clinical suspicion. After hospital discharge, half of the 12 patients who survived continued to use immunosuppressive therapy.

**Figure 1:**
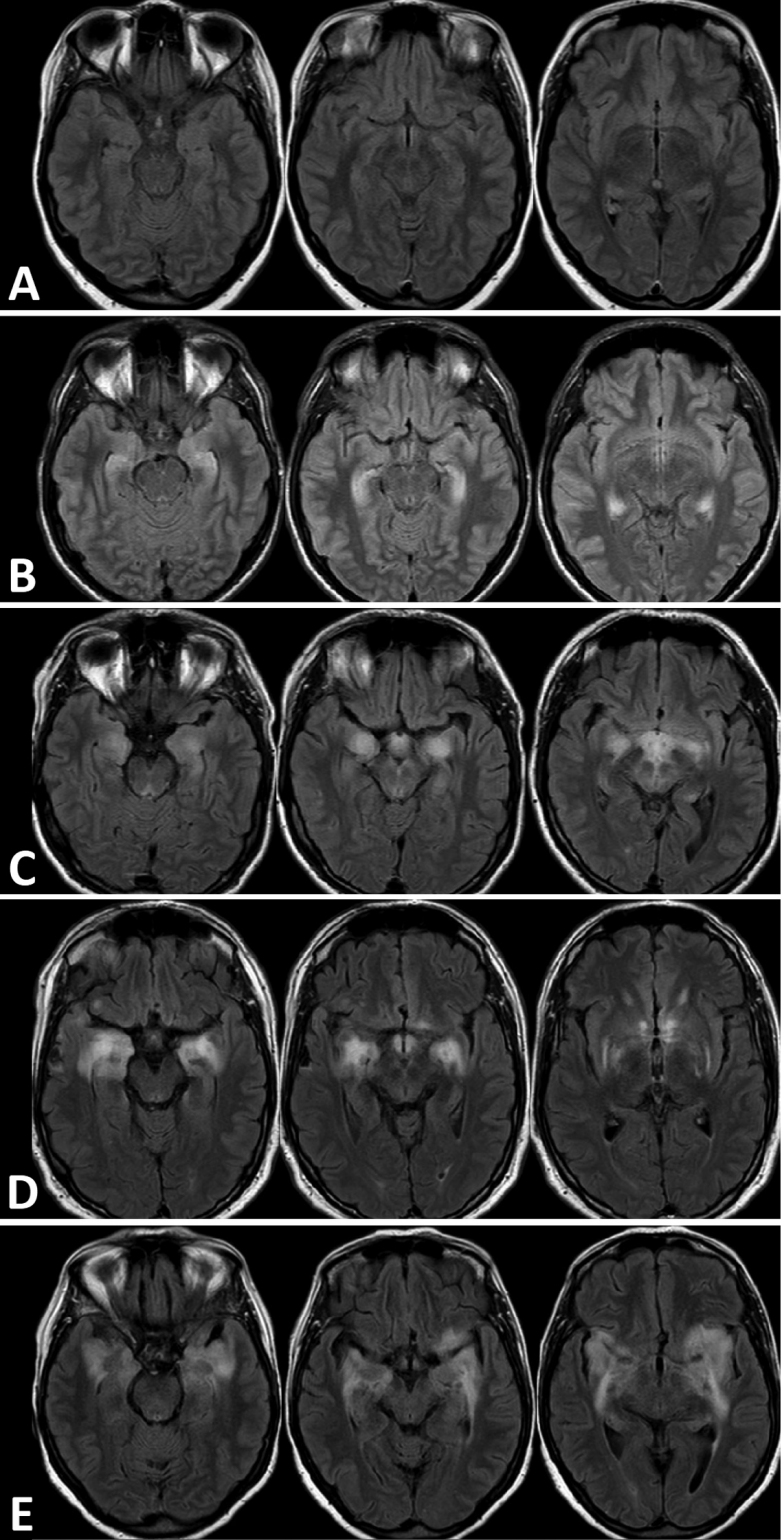
T2-weighted-fluid-attenuated inversion recovery (T2-FLAIR) brain magnetic resonance imaging (MRI) scans. ( **A** ) Encephalitis with no alterations on MRI (patients 2, 3, 4, 10 and 14). ( **B** ) Limbic encephalitis (patients 5, 6, 7, 8, 9, and 15). ( **C** ) Temporal lobe and diencephalon involvement (patient 1). ( **D** ) Temporal lobe, diencephalon and basal ganglia involvement (patient 13). ( **E** ) Temporal lobe, insula and basal ganglia involvement (patients 11, 12, 16, 17).

All patients were evaluated with EEG, CSF examination, and MRI. Neoplasia was identified in only one patient (patient 1), namely an embryogenic testicular carcinoma. The patients' mean CSF protein level was 32 (±30.46) mg/dL and most of them had lymphocytic pleocytosis (15 patients, 88.2%), with a mean total leukocyte count of 19 (±27.04)/μl. Status epilepticus was diagnosed during the acute phase in 12 patients (70.5%). All 7 survivors remained with epilepsy, and 4 developed refractory epilepsy during follow-up. In regards to the first MRI performed on admission ( Figure 1 ), bilateral T2-weighted-fluid-attenuated inversion recovery (T2-FLAIR) hyperintensity in the mesial temporal lobe was detected in 11 patients (64.7%). In 6 patients, including all patients positive for anti-GAD antibodies, the signal was highly restricted to this area. On the other hand, no MRI abnormalities were detected in any of the anti-NMDAr patients.

Due to clinical complications related to encephalitis, 5 deaths occurred during hospitalization (patients 2, 3, 4, 14, and 15) and 1 during follow-up (patient 1). All patients diagnosed with definite anti-NMDAr encephalitis died during diagnostic hospitalization. Patient 1 died after complications of disseminated neoplasia; patients 2, 4, 14, and 15 as a result of infectious complications; patient 3 due to massive pulmonary thromboembolism. Follow-up period of hospital survivors ranged from 2 to 72 months, with a mean value of 23 [ ± 21.08] months. The majority became functionally dependent (8 patients, 72.7%), with a mRS of 3 [ ± 1.57].

The mean time interval between diagnostic suspicion and definitive diagnosis was 15 [ ± 15.8] days, and it varied according to the institution's authorization to perform antibody testing. When the institution authorized antibody testing immediately, the interval between suspicion and diagnosis averaged 9 [ ± 6.9] days. When initially denied, the average time was 32.5 [ ± 12.4] days. The mean time from diagnostic suspicion to therapeutic initiation was 2 [ ± 3.4] days. A major limiting factor for the non-immediately starting of immunotherapy was the possibility of an underlying viral or bacterial infection. Most patients required admission to an intensive care unit (ICU) (70.5%), mainly due to status epilepticus. The mean total length of stay was 37 [ ± 52,2] days, whilst the mean length of stay in the ICU was 17 [ ± 28,6] days. It is noteworthy that all patients diagnosed with status epilepticus were evaluated with EEG followed by electroencephalographic monitoring, which made it possible to unequivocally establish this diagnosis.

## DISCUSSION

This case series presents 17 patients with newly diagnosed AE who had worse clinical outcomes than those described in the literature. In our sample, behavioral symptoms were the most prominent ones at disease onset, and clinical outcomes were mostly poor. Our sample included young adults or middle-aged patients younger than 50 years, except for 3 patients, which is consistent with previous data. 11 Antibodies were not available for all patients due to the resource-limited setting.

In our sample, anti-NMDAr encephalitis was diagnosed in 3 patients, specifically a 21, 31, and 42-year-old woman with no previous psychiatric comorbidity who presented with symptoms of behavioral change (patient 2: emotional lability; patient 3: a manic episode that progressed to catatonia; patient 4: aggressiveness and drowsiness), which started between 7 and 14 days before diagnostic admission. Despite the known relationship with ovarian teratoma, 12 neoplasm investigation was negative for all three. These patients presented no abnormalities on the MRI scan. This is the case for about half of the cases of anti-NMDAr encephalitis described in the literature. 13 During hospitalization, all patients evolved with refractory status epilepticus, thus requiring intensive care, and thereafter died from related complications. Despite the clinical severity, the mortality of anti-NMDAr encephalitis ranges from 5 to 11% in the literature. 14 15 Among the factors that may justify the negative outcome in our sample, delay in the diagnostic recognition and initiation of therapy are of major importance. Two of the patients were initially hospitalized in psychiatric units. Two of them also only received the definitive diagnosis post-mortem due to the unavailability of tests for anti-NMDAr antibodies in our institution. Owing to anti-NMDAr encephalitis, the most commonly described form of AE, seizure was the most frequent symptom (81.2%), followed by psychosis (70.5%) and cognitive impairment (47%). Tumor associations were relatively uncommon (22.1%) and on average 93.1% of cases received immune-modulating treatment. Clinical outcomes could be ascertained in 22 studies representing 1294 patients. A good outcome was demonstrated in 72.6% of cases, depending on the study and follow-up timeframe. 16

In patients with clinical suspicion of encephalitis, the detection of anti-GAD antibodies in the CSF or high serum titers (> 1000 IU/ml) was accepted as significant for correlation with the neurological syndrome. 5 In our sample, we identified 4 patients with this autoantibody, 3 of whom with serum detection and 1 with CSF detection (patient 5, who also presented anti-VGKC in the CSF). The identification of anti-VGKC may not have clinical significance, as more recent research has demonstrated that the target antigen is directed to specific cell membrane proteins LG1 and CASPR2, called the anti-VGKC5 complex 5 ; this is a limitation of our study.

Patient 15 had been diagnosed with anti-GAD cerebellar ataxia seven years earlier, receiving corticosteroid therapy and rituximab in the first 3 years of the disease, but later suspended because of a lack of relevant therapeutic effect. Clinically, all patients presented with behavioral alteration and memory deficit, and only patient 15 had a concomitant epileptic seizure in the initial presentation of encephalitis. Interestingly, in addition to the cognitive impairment, patient 9 presented with Guillain Barré-like syndrome with ascending flaccid tetraparesis, areflexia, and dysautonomia, preceded by a viral prodrome (viral parotitis) 20 days earlier. In spite of the probable spurious association, and although extremely rare, some case reports link the anti-GAD autoantibody with Guillain Barré syndrome and its variants. 17 18 During the acute course of the disease, all patients had epileptic seizures and 3 of them developed status epilepticus, requiring ICU care. However, this type of encephalitis rarely leads to status epilepticus. 19 After a thorough evaluation, these patients were diagnosed with definite autoimmune limbic encephalitis, a pattern known to be related to anti-GAD-and anti-VGKC complex autoantibodies. 19 20 Another two patients were diagnosed with definite autoimmune limbic encephalitis without the identification of autoantibodies. For these cases, anti-NMDAr antibodies were negative.

Regarding the identification of occult neoplasia, only one patient was diagnosed with paraneoplastic encephalitis (patient 1). This patient presented with a limbic-diencephalic syndrome with predominant symptoms of impaired working memory, altered sleep-wake cycle, and hyperphagia, which led to the identification of anti-Ma2 autoantibody, with the posterior diagnosis of embryonic testicular carcinoma. Dalmau et al. (2004) 21 described a series of cases in which this autoantibody was most often related to testicular cancer and limbic, diencephalic, and brainstem encephalitis. Although other onco-neural autoantibodies were identified, such as anti-Hu, related to more than 80% of the cases of paraneoplasia, 22 no other neoplasms were identified in our patients.

Epileptic seizures are frequent manifestations of AE and provide greater morbidity to this condition. Conceptually, when they occur in the active phase of the disease, they are named acute symptomatic seizures and usually resolve with appropriate early immunotherapy. However, some patients remain with epileptic seizures due to related autoimmune factors, encephalitis sequelae, or a combination of both. This can lead to a chronic brain disorder, known as autoimmune-associated epilepsy, for which immunotherapy is often ineffective. 23

**Figure 2:**
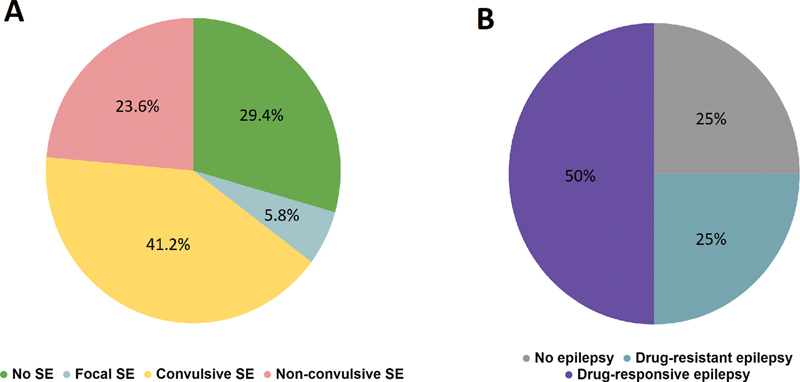
Abbreviation: SE, status epilepticus. Proportions of patients evolving with status epilepticus during hospitalization ( **A** ) and epilepsy during outpatient follow-up ( **B** ).

In our patients, although only 29.4% of them initially presented with an epileptic seizure, 82% had an acute symptomatic seizure related to AE during hospitalization, and 70% developed status epilepticus ( Figure 2A ). In a retrospective cohort study, Zhang et al. (2019) 24 stated that epileptic seizures occurred in most of the patients during the acute phase of the disease. This was one of the initial symptoms of the condition for up to 70% of them. In anti-NMDAr encephalitis, Liu et al. (2017) 25 pointed out that 80% of their patients had an acute symptomatic crisis, but only half evolved to status epilepticus. In a retrospective cohort focused on patients with AE requiring ICU care, Schubert et al. (2019) 26 described that 35% of them evolved with status epilepticus. Similarly, in another observational study of AE patients with severe neurological dysfunction, Wang et al. (2022) 27 declared that 40% of them were admitted to ICU because of status epilepticus. In our sample, 41.6% of the patients who presented with status epilepticus died during the hospitalization, which is also greater than what is found in the literature. 28 29

During outpatient follow-up, 75% of surviving patients developed epilepsy and half of them became drug-resistant (i.e., failed to control seizures after at least two proper drugs in adequate doses), as shown in Figure 2B . Although some case series showed a lower prevalence of epilepsy at follow-up visits, 23 24 only patients with encephalitis related to antibodies against an intracellular antigen or patients without autoantibody identification survived in our sample, conditions associated with a worse prognosis. 16 23 In a retrospective cohort of temporal lobe epilepsy patients with anti-GAD limbic encephalitis presentation, Joubert et al. (2020) 30 stated that only 33% were seizure-free at the end of outpatient follow-up (average of 49 months). Likewise, Falip et al. (2020) 31 described in a prospective study that 70% of patients with anti-GAD autoimmune-related epilepsy became drug-resistant during follow-up. In relation to autoantibody-negative AE, Von Rhein et al. (2016) 32 demonstrated that only 46% were seizure-free after a median follow-up of 18 months. This retrospective cohort encompassed antibody-negative encephalitis patients with recent-onset temporal lobe epilepsy.

The prognosis related to the different subtypes of AE is variable, and patients with cell surface antibodies have a better outcome than those with target intracellular antibodies. 16 33 Those who receive earlier immunotherapy probably have a better outcome as well. 16 33 Other factors may influence the prognosis despite inconsistencies among studies, such as early identification, autonomic dysfunctions, MRI and CSF abnormalities, presence of status epilepticus, need for ICU admission, and mechanical ventilation. 16 34 35 36 A better understanding of these factors and their association with the prognosis of AE can influence future therapeutic decisions and improve care for this disease.

**Figure 3:**
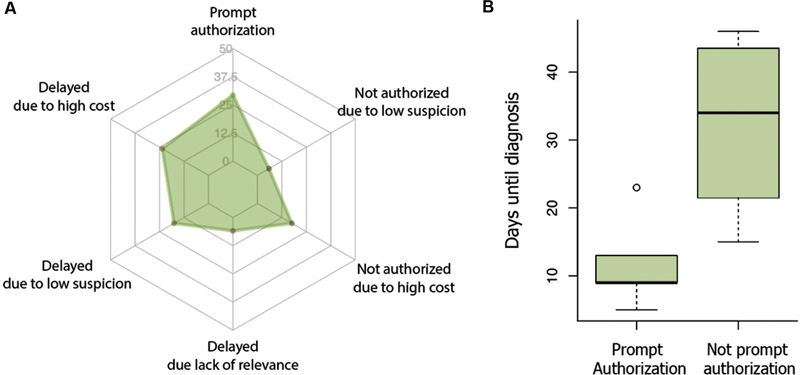
( **A** ) Reasons for delaying or not performing autoantibodies tests. ( **B** ) Time difference to diagnosis between groups with promptly authorization and not promptly authorization.

Time to diagnosis was severely impacted by the institution's authorization of autoantibody testing in our sample. When immediately authorized, the timing was three times shorter than when initially denied. Notably, early and aggressive treatment is imperative to improve clinical outcomes in AE patients ^5.^ It is important to mention that these patients were admitted to a public hospital. Therefore, privately insured patients were not included in this setting. Among the main reasons for the refusal of autoantibody testing, there are the high related costs and the need to exclude alternative diagnoses before ordering the tests ( Figure 3 ). Neuronal autoantibody testing is expensive and its public availability is limited even in rich countries. 1

Despite the delay in definitive diagnosis, there was no significant delay in starting empiric immunotherapy treatment. On the other hand, the lack of a precise diagnosis may have influenced the choice of first- and second-line immunotherapy treatment, and–consequently– the functional outcome. DeSena and colleagues demonstrated in a small retrospective study that patients with anti-NMDAr autoimmune encephalitis had a better functional outcome when treated with corticosteroid therapy followed by plasma exchange, compared to those treated with corticosteroid therapy alone. 37 In our series, of the three patients identified with anti-NMDAr, only one received plasmapheresis. When treatment fails 2 to 4 weeks after starting immunotherapy, the use of second-line therapy is suggested, such as rituximab and cyclophosphamide. 1 Rituximab appears to be more effective in patients with antibody-mediated autoimmune encephalitis, such as anti-NMDAr, while cyclophosphamide appears to be more effective in patients with cell-mediated autoimmune encephalitis, such as paraneoplastic syndromes. 1 In our sample, only 3 patients received any of these second-line therapies. In addition to the related high economic cost, the lack of a definitive diagnosis may have influenced the difficulty in obtaining medication.

AE is a serious condition that must be recognized and treated early. In our sample size, only a minority of patients (3 patients, 17.6%) acquired functional independence after discharge. Among other patients, a third remained functionally dependent but walking without assistance (6 patients with mRs 3, 35.2%), and a third died in the hospital (6 patients, 35.2%). Functional outcomes reported in this study were worse than those described in the literature. 16 24 27 Even if only patients diagnosed and treated in Latin America were taken into account, most were able to achieve functional independence at follow-up, 17 which differs significantly from our sample. We believe that these worse outcomes are related to the identification of autoantibodies against intracellular antigen in a significant portion of the sample, a condition related to the lower response to immunotherapy. Moreover, the high rate of status epilepticus and need for ICU, as well as the lower use of second-line immunosuppressive therapies, may have significantly influenced the outcome of these patients. Finally, the economic difficulties encountered in both the diagnostic and therapeutic processes and the lack of an institutional protocol to standardize decisions may have negatively impacted the final result.

Despite our efforts, this study was also subject to bias. Individuals were selected according to clinical suspicion, and those cases that were not clinically relevant may have been potentially overlooked. Individuals with rapidly progressive clinical course may also be subject to other clinical hypotheses. Authorization by the financial staff may present a challenge to systematically reproduce other institutions, though this is a reality throughout the public health system worldwide. 38

In conclusion, in this resource-limited setting, individuals with AE had a worse clinical outcome than described in the literature. In our sample, we found a large number of patients with autoantibodies against intracellular antigens, who developed status epilepticus and required ICU care, as well as presented an unsatisfactory response to the established immunotherapy; these factors were probably preponderant in the negative results found. The financial difficulties encountered are mainly related to authorization for autoantibody testing, which impacted the definitive diagnosis and, thus, the use of second-line therapies. In brief, mortality occurred at a greater rate, functional outcomes were inferior and epilepsy developed more often.
